# Antibiotics Prescribing in Intensive Care Settings during the COVID-19 Era: A Systematic Review

**DOI:** 10.3390/antibiotics10080935

**Published:** 2021-08-02

**Authors:** Lubna I. Abu-Rub, Hana A. Abdelrahman, Al-Reem A. Johar, Hashim A. Alhussain, Hamad Abdel Hadi, Nahla O. Eltai

**Affiliations:** 1 Biomedical Research Center, Qatar University, Doha 2713, Qatar; lubna.ali@qu.edu.qa (L.I.A.-R.); hana.abdelrahman@qu.edu.qa (H.A.A.); h.alhussain@qu.edu.qa (H.A.A.); 2 Barzan Holdings, Doha 7178, Qatar; ajohar@barzanholdings.com; 3 Communicable Diseases Centre, Infectious Disease Division, Hamad Medical Corporation, Doha 3050, Qatar; HAbdelHadi@hamad.qa

**Keywords:** COVID-19, SARS-CoV-2, antibiotic, antibiotic prescription, ICU, bacterial co-infections

## Abstract

The prevalence of patients admitted to intensive care units (ICUs) with SARS-CoV-2 infection who were prescribed antibiotics is undetermined and might contribute to the increased global antibiotic resistance. This systematic review evaluates the prevalence of antibiotic prescribing in patients admitted to ICUs with SARS-CoV-2 infection using PRISMA guidelines. We searched and scrutinized results from PubMed and ScienceDirect databases for published literature restricted to the English language up to 11 May 2021. In addition, we included observational studies of humans with laboratory-confirmed SARS-CoV-2 infection, clinical characteristics, and antibiotics prescribed for ICU patients with SARS-CoV-2 infections. A total of 361 studies were identified, but only 38 were included in the final analysis. Antibiotic prescribing data were available from 2715 patients, of which prevalence of 71% was reported in old age patients with a mean age of 62.7 years. From the reported studies, third generation cephalosporin had the highest frequency amongst reviewed studies (36.8%) followed by azithromycin (34.2%). The estimated bacterial infection in 12 reported studies was 30.8% produced by 15 different bacterial species, and *S. aureus* recorded the highest bacterial infection (75%). The fundamental outcomes were the prevalence of ICU COVID-19 patients prescribed antibiotics stratified by age, type of antibiotics prescribed, and the presence of co-infections and comorbidities. In conclusion, more than half of ICU patients with SARS-CoV-2 infection received antibiotics, and prescribing is significantly higher than the estimated frequency of identified bacterial co-infection.

## 1. Introduction

In December 2019, an outbreak of novel coronavirus was first detected in Wuhan City, the capital of Hubei province in Central China. The virus spread rapidly to other parts of the world, and by 11 March 2020, COVID-19, as it is commonly known, was declared a pandemic by the World Health Organization (WHO) [1]. Statistics from the WHO indicate that as of May 2021, more than 165 million persons were infected with COVID-19, and more than 3 million patients died [2]. The ambiguity of this serious disease over the first wave swept worldwide, posing considerable challenges, including diagnosis, management, provision of resources, and ethical consideration to all global healthcare institutions. Therefore, the catastrophic situation resulted in many patients with a wide range of clinical manifestations ranging from asymptomatic to fatal diseases that required critical care, including ICU admission [3]. Furthermore, in critical care settings, research indicates that COVID-19 is associated with increased mortality of up to 50% with pre-existing comorbidities, such as diabetes, hypertension, and renal and cardiovascular complications, in critical care settings [4].

It is axiomatic that hospital admissions increase risks of healthcare-associated infections (HCAIs), leading to a noticeable increase in antibiotic consumption [5]. A recent study conducted on ICU patients in 88 countries highlighted that 70% of hospitalized patients receive at least one antibiotic during acute admission; of this cohort 54% developed a secondary bacterial infection that necessitated antibiotic therapy [6]. In patients with severe disease, the WHO recommends the provision of antimicrobial therapy to prevent furthers infection complications, leading to severe acute respiratory distress syndrome (ARDS) and multi-organ failure [7]. Despite that, scientific literature still has many gaps and uncertainties regarding the impact of antimicrobial therapy during the pandemic, particularly in critical care settings, which needs to be assessed and evaluated for the sake of global health as well as humanity. Therefore, this review aims to determine the prevalence of COVID-19 patients admitted to the ICUs that have been exposed to antimicrobial therapy correlated against isolated pathogens, comorbidities, and clinical outcomes.

## 2. Results

### 2.1. Outcome of Study Identification Process

**Figure 1:**
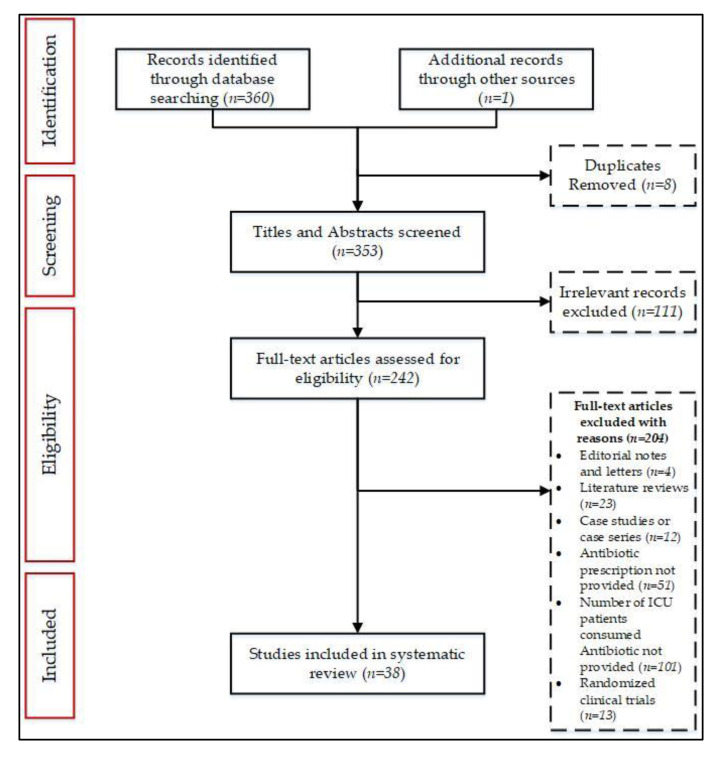
PRISMA schematic selection process of the included studies at each stage of the screening process.

A total of 361 records were identified from databases and manual searches. After removing duplicates (*n* = 8), titles and abstracts were screened for 353 studies, and 242 short-listed articles were eligible for full-text evaluation. Of the 242 full-text studies, 38 were included in the review. Figure 1 provides details of excluded records at each stage of the screening process.

### 2.2. Characteristics of 38 Included Studies

Of the 38 included studies in this review, the majority were from countries significantly affected by the COVID-19 pandemic: Six studies each (15.8%) were from USA, China, and France. Three studies each (7.9%) were from Saudi Arabia (KSA) and Italy. Two additional studies each (5.3%) were from Iran, Spain, and South Korea. The remaining studies were from Brazil, Oman, Congo, Turkey, UK, Colombia, Kosovo, and Indonesia. Detailed data on the characteristics of the conclusive studies are given in Table S1 [8,9,10,11,12,13,14,15,16,17,18,19,20,21,22,23,24,25,26,27,28,29,30,31,32,33,34,35,36,37,38,39,40,41,42,43,44,45].

#### 2.2.1. Patient Characteristics

In total, 2715 patients were reported from the 38 studies included in this review, of which 71% received antibiotics during their ICU stay (1929/2715). Demographic data of the study population included both genders with a male/female ratio of 1.7:1. We also included all ages ranging from neonates, children, adults, and old age patients, encompassing pregnant women, patients with cancer, and immunocompromised patients. Based on 17 studies, the average median age of patients was 51.8 years, while the average mean age of patients was 62.7 years based on six studies since age was not specified in the remaining 15 studies. The average length of stay at ICUs ranged between 7.7 and 20 days for the studies’ median and mean numbers with a standard deviation of (4.57, 12.46), respectively (see Table S1).

#### 2.2.2. Associated Comorbidities

**Figure 2:**
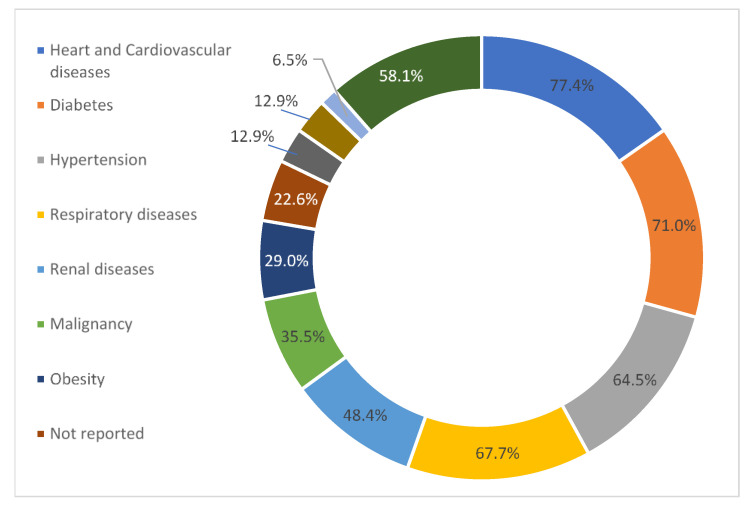
Percentage of associated underlying conditions reported based on 31 reviewed studies. Data on comorbidity were not specified among the seven included studies.

Heart and cardiovascular diseases, hypertension, diabetes mellitus, and respiratory diseases were commonly recorded as comorbidities among 31 included studies, whereas seven remaining studies did not report any comorbidities. In addition, obesity; malignancy; liver, renal, and neurological diseases; and organ transplantation (kidney, lung, liver, and heart) were moderately prevalent according to the reviewed studies (Figure 2).

#### 2.2.3. Antibiotic Prescription

Of the analyzed studies, 52.6% (*n* = 20) reported prescribing specific antimicrobial to all patients (100%), while 86.8% (*n* = 33) recorded prescribing antibiotics to over 50% of the patients. From the prescribed antibiotics, third generation cephalosporin had the highest frequency amongst reviewed studies (36.8%) followed by azithromycin (34.2%). In addition, 10.5% of the included studies reported a moderate prescription of amoxicillin-clavulanate, piperacillin-tazobactam, and meropenem. One type of each antibiotic (2.6%) was prescribed to patients admitted to ICUs: amoxicillin, beta lactam-beta lactamase inhibitor, imipenem, moxifloxacin, aminoglycosides, trimethoprim-sulfonamide, lincosamides, tigecycline, and daptomycin. Data on the type of antibiotics were not specified for 42.1% of reporting studies (Table 1).

#### 2.2.4. Bacterial Co-Infection

**Figure 3:**
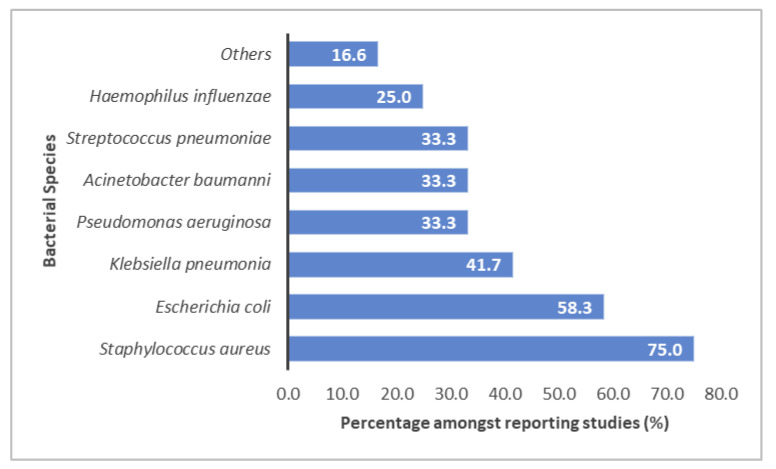
Percentage of reported bacterial species in ICU patients with COVID-19 from reviewed studies (*n* = 12/38). “Others” includes the organisms *Morganella morgani*, *Mycoplasma pneumoniae*, *Moraxella catarrhalis, Stenotrophomonas, Burkholderia gladiol, Pneumococcus pneumoniae*, and *Legionella pneumophila*.

Of all reported studies, only 30.8% discussed the presence of bacterial co-infection, of which 15 different bacterial species were reported. From evaluated studies, co-infection with *Staphylococcus aureus* (*S. aureus)* was the highest (75%), followed by *Escherichia coli* (*E. coli*) (58.3%), *Klebsiella pneumonia* (41.6%), then *Pseudomonas aeruginosa* (33.3%). Furthermore, 25% of the studies recorded other pathogens such as *Acinetobacter baumannii*, *Streptococcus pneumonia,* and *Haemophilus influenza* (Figure 3). On the other hand, no clear data on bacterial co-infection were reported in most reviewed studies (69.2%).

#### 2.2.5. Death Rate

Antibiotic exposure and mortality rates were one of critical analysis outcomes throughout the review. Evaluated data from the reviewed studies demonstrated strong positive Pearson correlation coefficients (r = 0.925), between admission to ICU and exposure to antibiotic treatment, which are statistically significant (*p* < 0.001). In addition, a moderate Pearson positive correlation coefficient (r = 0.393) was recorded between prescribing antimicrobial and the outcome of patient death, which is statistically significant, *p* = 0.029.

## 3. Discussion

The unprecedented recorded numbers of patients infected with SARS-CoV-2 continues to escalate worldwide. As observed from the early stages of the pandemic, the extensive practice of prescribing antimicrobials for the treatment of COVID-19 infected patients might lead to the increased adverse events and long-term consequences such as antimicrobial resistance [46]. Several studies confirm that most hospitalized patients with COVID-19 are managed with broad-spectrum antimicrobials with unproven efficacy [47]. Cong et al. (2021) highlighted that the percentage of antibiotic prescriptions for patients with both severe/critical disease nearly equaled the percentage for patients with mild/moderate disease, even though more critically ill patients are at greater risk of developing secondary bacterial infections [3]. These unnecessary antimicrobials and excessive prescribing for mild and moderate COVID-19 will probably increase risks for adverse events and selective development of multidrug-resistant bacterial pathogens at local healthcare and regional levels.

To our knowledge, the presented study is the first systematic review to evaluate the prevalence of antibiotic prescribing for patients admitted to ICU settings with confirmed SARS-CoV-2 infection. Our review involved 16 different countries, including major countries, where the plight of the pandemic was evident: China, USA, Italy, and France. The demographic data of this review demonstrate that 2715 ICU patients with ages ranging from 1 day to 92 years and a mean age of 62.7 were exposed to antibiotic therapy. Analogous to our findings, many previous studies concluded that old age patients are more susceptible to severe and critical illness leading to ICU admission. In addition to the clear age-related morbidity and mortality, older patients are more prone to an increase of long-term complications [48,49]. It is relevant to mention that our results were similar to those of Peckham H. et al. (2020) [50], who found that the proportion of male admission to ICUs was significantly higher when compared to females. These highlighted numbers can play an important role in risk stratifications and implications for the clinical management of COVID-19 [50].

Our reviewed studies of ICU patients with COVID-19 revealed associated underlying conditions including hypertension; diabetes mellitus; obesity; malignancy; heart, cardiovascular, liver, respiratory, renal, and neurological diseases; cancer; and organ transplantation. From early observational studies during the pandemic, several studies reported increased incidence and severity of COVID-19 in patients with similar underlying premorbid conditions [51,52,53,54]. Conversely, recorded severity markers include acute kidney and liver injuries, demonstrating the potential contribution of antibiotics exposure in vulnerable populations [54,55,56,57].

The review demonstrated the significant global healthcare hospital prescribing of antimicrobials, which might be a temporary pattern associated with the serious pandemic. Since the first wave of this pandemic, multiple regional protocols included empirical antimicrobials such as ceftriaxone and azithromycin, leading to a substantial increase in antimicrobial consumption at various healthcare settings [36,37]. In accordance, based on our findings, third generation cephalosporin (36.8%) and azithromycin (34.2%) antibiotics were the most prevalent antimicrobial agents reported during the management of COVID-19 in patients admitted to ICUs (Table 1). Macrolide azithromycin and hydroxychloroquine intended for the management of COVID-19 during the pandemic were initially used as an adjuvant anti-inflammatory and antiviral therapy rather than for their antibacterial properties. Nevertheless, conflicting studies and reports delayed their removal from management protocols [58,59]. Although ambiguous, the doors remain open for an antimicrobial therapy for various stages of the mysterious disease. A recent study constructed a computational drug repurposing approach in an attempt to find existing drugs against COVID-19, advocating that cephalosporin, ceftaroline, fosamil have a remarkable effect in treating vascular complications in infected patients.

Moreover, these antimicrobials act as antibacterial agents against MRSA, which is the most abundant observed bacterial infection reported in this review [58]. Of note, excessive use of these two antimicrobials probably contributed to some adverse outcome scenarios. They have been associated with cardiac toxicities, including arrhythmias and sudden death in susceptible patients with underlying cardiac diseases [59].

Consequently, the latest updates from the WHO’s guidance on the clinical management of COVID-19 states that antibiotic overuse increases the risk of emergence and transmission of multidrug-resistant organisms (MDROs). Therefore, infection rates with MDROs are more challenging to treat, coupled with increased mortality of acute COVID-19 cases and increased management costs [60]. Worryingly, this review determines a moderate positive correlation (r = 0.393) between prescribing antibiotics and the percentage of ICU patient death, which is statistically significant (*p* = 0.029). This can be evaluated as an observation rather than causation since more antimicrobials were prescribed for patients with critical disease, which correlated with increased mortality. Nevertheless, more detailed evaluations are needed mainly for associated adverse events such as antimicrobial-induced acute kidney injuries in critical care settings [61]. Moreover, there is a difference in reporting mortality rates from previously reviewed studies covering a wide range between 0% and 100% of the ICU patients [28,34,45] (see Table S1). This mortality rate variation appeared to be affected by various clinical and social factors, such as age, comorbidities, regional COVID-19 pandemic situation, and timely access to optimal healthcare [20].

Of note, a wide range of broad-spectrum antibiotics has been frequently prescribed for ICU patients, including piperacillin-tazobactam, meropenem, amoxicillin, beta lactam-beta lactamase inhibitor, imipenem, moxifloxacin, aminoglycosides, trimethoprim-sulfonamide, lincosamides, tigecycline, and daptomycin, highlighting potential further development of current or future AMR as most of them have been prescribed empirically or as prophylaxis to prevent secondary bacterial infection [62]. In support of this, our review revealed that most of the studies (69.2%) demonstrated the spectrum of prescribing antibiotics for patients admitted to ICUs with COVID-19 lacking any clear evidence of bacterial co-infection. Despite that, 15 different bacterial species were identified in 30.8% of the reviewed studies. Our findings support the results of a multi-hospital cohort study in the USA [62], which similarly showed widespread use of antibiotics. The prescription of early empiric antibacterial therapy treatment occurred in 56.6% of 1705 patients, in which only 3.5% of the cases were confirmed with bacterial infection [63].

The observed high rates of prescribing antimicrobial therapy to COVID-19 patients admitted to ICUs can be partially understandable, considering the complexity of the novel critical illness and challenges in excluding associated bacterial co-infections [64].

With limited accurate tests for pathogen identifications, Nag and Kaur, (2021) [65] reported that the incidence of superadded infection ranged between 13.5% and 44% for patients with COVID-19 admitted to the intensive care unit (ICU), usually ventilator-associated pneumonia (VAP) caused by bacterial or fungal causes [65]. Another group reviewed the earliest SARS-CoV-2 pandemic cases, and their study observed that overall, 14% of the hospitalized COVID-19 ICU patients had a bacterial co-infection [66]. In the same study, the authors compared influenza and COVID-19 pandemic patients and found that bacterial co-infections were more prevalent in influenza patients than in COVID-19 patients. This bacterial infection was associated with the high mortality rate of influenza A (H1N1) [67]. Commonly, the identification of co-infection bacteria is consistent with the types of pathogens usually associated with hospital-acquired pneumonia (HAP) or ICU-HAP as a complication of ICU and does not advocate a specific preference for bacterial co-infections in COVID-19 [67].

Intriguingly, for patients with COVID-19 admitted to ICUs, pathogen identification revealed the dominance of the Gram-positive *S. aureus* infection in 75% of studies as opposed to the usual culprits of Gram-negative bacteria (GNB). This observation emulates the previous noticeable scientific and clinical knowledge of secondary *S. aureus* pneumonia following influenza infection attributed to upregulation of specific *S. aureus* virulence factors [68].

A comparable result found in a study reported the rate of bacterial co-infection at ICU patients with SARS-CoV-2 pneumonia, which was about 28%; generally, it was associated with *S. aureus, H. influenzae, S. pneumoniae,* and Enterobacteriaceae [27]. Likewise, during the SARS-CoV-2 outbreak (2003), there was a notable increase in *S. aureus* superinfection [69]. Clinicians should be alert to the high percentage of *S. aureus* co-infection during COVID-19 pneumonia [26]. This significant analyzed outcome must be considered when deciding empirical antimicrobial therapy that ideally should include *S. aureus* cover as superior or equal to GNB.

Although there were limited data in the reviewed studies about the antimicrobial resistance patterns in the identified bacteria, a study in our review reported the detection of extended-spectrum beta-lactamase (ESBL) *E. coli* in ICU patients [19]. Furthermore, a retrospective report in France mentioned the detection of other MDR bacteria such as *Morganella morganii*, *Enterobacter cloacae*, *Stenotrophomonas maltophilia*, *Enterobacter cloacae*, *Klebsiella pneumoniae,* and *E. coli* [37]. Some studies also reported detection of methicillin-resistant *S. aureus* (MRSA) with different percentages: 1.9%, 5%, and 61.5% among ICU COVID-19 patients [13,26,44]. Compared with the SARS pandemic, the rate of isolating MRSA in the ICU patients’ samples was 3.53% before the SARS pandemic; then, it increased to 25.3% during the SARS pandemic [70]. These findings emphasize secondary MDRO infections in patients due to *Acinetobacter baumannii* and *S. aureus* resistant to multiple commonly used antibiotics [13]. In developing countries, where there are well-known high rates of multidrug-resistant organisms in ICU settings, superinfections in COVID-19 patients can cause a huge challenge leading to an upsurge in mortality [64]. Correspondingly, there is a necessity to link empirical antibiotics therapy at ICU settings based on local antibiogram patterns in countries with a high burden of the disease [46]. Concomitantly, effective antibiotic stewardship should be applied in ICU settings, which has a critical role in preventing the inappropriate use of antimicrobials. Nevertheless, data related to antibiotic prescribing in the ICU for COVID-19 patients are limited but continue to emerge; hence, more studies should be focused on this crucial aspect of critical care management.

Understanding how long patients hospitalized with COVID-19 remain in ICU is critical to studying the associated comorbidities. ICU length of stay (LoS) was reported in 16 studies and varied from 1 day to 55 days with an average median value of 7.7 (SD 4.57). These findings are similar to those of the review by Rees E.M. et al. (2020) that compared the LoS for ICUs in China and outside China and found a median value of 8 days (IQR 5–13) for China and 7 (IQR 4–11) days outside of China [71]. This review reports that most COVID-19 ICU patients were from the older population, which explains the observed extended stay supported by two previous studies that concluded that critically ill patients of an older age group tend to have longer LoS [72,73].

Overall, during the COVID-19 pandemic, there has been a significant use of antibiotics and a wide range of specific antibiotic prescribing for COVID-19 patients in ICU, notably given to old age patients with existing underlying comorbidities, despite the paucity of evidence of associated bacterial infections.

Despite many positive conclusive outcomes, the presented review might have some limitations that influence the overall quality of evaluated data. Restricting search criteria from only limited databases published in English might not capture all relevant studies in the subject. In addition, although almost 400 studies were primarily identified, only 38 had the needed inclusion criteria, and some of which lacked needed additional details such as types of prescribed antimicrobials. Nevertheless, the review covered a crucial area of patients with COVID-19 during their admission to critical care, raising many valid observed points, which warrant further exploration.

## 4. Materials and Methods

We conducted this systematic review based on PRISMA guidelines for systematic reviews [74] to determine the percentage of ICU patients diagnosed with COVID-19 who received antibiotics during their stay in the ICU. This is referred to as the prevalence of antibiotic use in ICU patients with established SARS-CoV-2 infection.

### 4.1. Search Criteria, Inclusion, and Exclusion

A literature search was accomplished through PubMed and Science Direct databases for published studies, using the keywords of “COVID-19’’, “SARS-CoV-2”, “antibiotics” and “ICU” for published literature in the English language.

All stated keywords were combined using the “OR” operator and “AND” operator for searching the literature. An additional article was included following a search of the Google Scholar search engine.

### 4.2. Inclusion Criteria

Articles that met our criteria were involved in our study: (1) observational studies of humans with laboratory-confirmed SARS-CoV-2 infection, clinical characteristics, and outcomes of antibiotics prescribed for ICU patients with SARS-CoV-2 infections, and (2) articles published between 1 December 2019 to 11 May 2021 in peer-reviewed journals.

### 4.3. Exclusion Criteria

The exclusion criteria were as follows: (1) articles that did not report data on the number and percentage of patients receiving antibiotics in the ICU; (2) articles with fewer than 10 patients (defined as case series) and/or case reports; and (3) randomized clinical trials, reviews, editorials, animal studies, letters, and conference abstracts (Figure 1).

### 4.4. Study Selection

Initial screening of titles and abstracts from the search was conducted by the author L.A. for the PubMed database and by L.A., H.A., and A.J. for the ScienceDirect database, and the authors identified studies that met all inclusion criteria and none of the exclusion criteria. All full-text studies meeting initial criteria were then reviewed by authors L.A., H.A., and A.J. for final inclusion in the review. Authors L.A. and H.A. analyzed 25% of the randomly selected studies for duplicate screening, and the author A.J. screened all selected studies for eligibility for quality assurance. Any disagreements were reviewed independently by another author who had not participated in the screening (H.A.A.).

### 4.5. Data Extraction

Authors L.A., H.A., and A.J. independently extracted data from included studies using a data collection form. We collected data on the following variables for demographics setting: country of study; healthcare setting (inpatient ICU vs. non-ICU); sample size; mean or median age; and proportion of male patients. Regarding clinical characteristics, we collected information on several ICU patients who were prescribed an antibiotic, the number of patients with comorbidities, and bacterial co-infections.

### 4.6. Data Synthesis and Analysis

The primary outcome was the prevalence of antibiotic prescribing among ICU patients with COVID-19. We assessed the number of patients prescribed an antibiotic at an ICU during their course of sickness while additionally observing the proportion of ICU patients that were positive to COVID-19. In the beginning, we classified by country to find variations in prescribing practices depending on geography. Then, we stratified by antibiotic prescribing. We also stratified prescribing by the bacterial infection identified with COVID-19 ICU patients in the study. Microsoft Excel 2016 and IBM^®^ SPSS^®^ Statistics v.26 were used for data analysis. The Pearson correlation test was used to correlate the different variants.

## 5. Conclusions

In conclusion, this review revealed a significant prescribing pattern of antibiotics in the ICU settings for COVID-19 patients with lower detection rates for pathogen identification coupled with the significant outcome of mortality. Since COVID-19 patients admitted ICUs were at the highest level of critical care, we strongly advocate a multidisciplinary approach for monitoring and managing appropriate and judicious prescribing of antimicrobials during ICU admissions throughout the pandemic. Assessment and patterns of antibiotic prescribing for COVID-19 ICU patients should support the identification of opportunities for timely interventions, refine antibiotic stewardship strategies to improve high-quality care, and ensure patient safety.

## Figures and Tables

**Table 1:** Percentage of the prevalence of prescribed antibiotics amongst reviewed studies.

Antibiotic	Frequency Amongst Included Studies	Percentage Amongst Reporting Studies (*n* = 38) (%)
**Cephalosporin**		
Third generation cephalosporin	14	36.8
Fourth generation cephalosporin	2	5.3
Cephalosporin (unspecified)	2	5.3
**Macrolides**		
Azithromycin	13	34.2
Macrolide (unspecified)	2	5.3
**Penicillin and Penicillin β-lactamase inhibitor combinations**		
Piperacillin-tazobactam	4	10.5
Amoxicillin-clavulanate	3	7.9
Penicillin	3	7.9
Amoxicillin	1	2.6
Beta lactam-beta lactamase inhibitor	1	2.6
**Carbapenems**		
Meropenem	4	10.5
Imipenem	1	2.6
Carbapenems (unspecified)	4	10.5
**Quinolones**		
Fluoroquinolones	3	7.9
Quinolones	2	5.3
Moxifloxacin	1	2.6
**Glycopeptide**		
Vancomycin	2	5.3
**Folate pathway inhibitors**		
Trimethoprim-sulfonamide	1	2.9
**Aminoglycosides**		
Aminoglycosides	1	2.9
**Lincosamides**		
Clindamycin	1	2.9
**Glycylcyclines**		
Tigecycline	1	2.9
**Lipopeptides**		
Daptomycin	1	2.9
**Others Antibiotic (unspecified)**	16	42.1

## Data Availability

The data presented in this study are available on request from the corresponding author.

## Supplementary Materials

The following are available online at https://www.mdpi.com/article/10.3390/antibiotics10080935/s1, Table S1: Demographical and clinical data of 38 studies related to antibiotics prescription in the ICU settings for COVID-19 patients.

## References

[1] Zhou F., Yu T., Du R., Fan G., Liu Y., Liu Z., Xiang J., Wang Y., Song B., Gu X. (2020). Clinical course and risk factors for mortality of adult inpatients with COVID-19 in Wuhan, China: A retrospective cohort study. Lancet.

[2] WHO Coronavirus (COVID-19) Dashboard WHO Coronavirus (COVID-19) Dashboard with Vaccination Data. https://covid19.who.int/.

[3] Cong W., Poudel A.N., Alhusein N., Wang H., Yao G., Lambert H. (2021). Antimicrobial use in COVID-19 patients in the first phase of the SARS-CoV-2 pandemic: Rapid review and evidence synthesis. medRxiv.

[4] Koeppen M., Rosenberger P., Magunia H. (2021). COVID-19 Related Cardiovascular Comorbidities and Complications in Critically Ill Patients: A Systematic Review and Meta-analysis. Clin. Med. Insights Circ. Respir. Pulm. Med..

[5] Langford B.J., So M., Raybardhan S., Leung V., Westwood D., MacFadden D.R., Soucy J.-P.R., Daneman N. (2020). Bacterial co-infection and secondary infection in patients with COVID-19: A living rapid review and meta-analysis. Clin. Microbiol. Infect..

[6] Rawson T.M., Moore L.S.P., Zhu N., Ranganathan N., Skolimowska K., Gilchrist M., Satta G., Cooke G., Holmes A. (2020). Bacterial and Fungal Co-infection in Individuals with Coronavirus: A Rapid Review to Support COVID-19 Antimicrobial Prescribing. Clin. Infect. Dis..

[7] Saleem Z., Godman B., Hassali M.A., Hashmi F.K., Azhar F., Rehman I.U. (2019). Point prevalence surveys of health-care-associated infections: A systematic review. Pathog. Glob. Health.

[8] Shekerdemian L.S., Burns J.P. (2021). Why Is Antibiotic Treatment Rarely Performed in COVID-19-Positive Children Admitted in Pediatric Intensive Care Units?-Reply. JAMA Pediatr..

[9] Faqihi F., Alharthy A., Alodat M., Kutsogiannis D.J., Brindley P.G., Karakitsos D. (2020). Therapeutic plasma exchange in adult critically ill patients with life-threatening SARS-CoV-2 disease: A pilot study. J. Crit. Care.

[10] Wang R., Kong L., Xu Q., Yang P., Wang X., Chen N., Li L., Jiang S., Lu X. (2021). On-ward participation of clinical pharmacists in a Chinese intensive care unit for patients with COVID-19: A retrospective, observational study. Res. Soc. Adm. Pharm..

[11] Lima-Setta F., de Magalhães-Barbosa M.C., Rodrigues-Santos G., das Figueiredo E.A.N., de Jacques M.L., de Zeitel R.S., Sapolnik R., da Borges C.T.S., Lanziotti V.S., de Castro R.E.V. (2021). Multisystem inflammatory syndrome in children (MIS-C) during SARS-CoV-2 pandemic in Brazil: A multicenter, prospective cohort study. J. Pediatr. (Rio J.).

[12] Maataoui N., Chemali L., Patrier J., Tran Dinh A., Le Fèvre L., Lortat-Jacob B., Marzouk M., d’Humières C., Rondinaud E., Ruppé E. (2021). Impact of rapid multiplex PCR on management of antibiotic therapy in COVID-19-positive patients hospitalized in intensive care unit. Eur. J. Clin. Microbiol. Infect. Dis..

[13] Sharifipour E., Shams S., Esmkhani M., Khodadadi J., Fotouhi-Ardakani R., Koohpaei A., Doosti Z., Ej Golzari S. (2020). Evaluation of bacterial co-infections of the respiratory tract in COVID-19 patients admitted to ICU. BMC Infect. Dis..

[14] Colmenero J., Rodríguez-Perálvarez M., Salcedo M., Arias-Milla A., Muñoz-Serrano A., Graus J., Nuño J., Gastaca M., Bustamante-Schneider J., Cachero A. (2021). Epidemiological pattern, incidence, and outcomes of COVID-19 in liver transplant patients. J. Hepatol..

[15] Yang L., Liu J., Zhang R., Li M., Li Z., Zhou X., Hu C., Tian F., Zhou F., Lei Y. (2020). Epidemiological and clinical features of 200 hospitalized patients with corona virus disease 2019 outside Wuhan, China: A descriptive study. J. Clin. Virol..

[16] Pereira M.R., Mohan S., Cohen D.J., Husain S.A., Dube G.K., Ratner L.E., Arcasoy S., Aversa M.M., Benvenuto L.J., Dadhania D.M. (2020). COVID-19 in solid organ transplant recipients: Initial report from the US epicenter. Am. J. Transplant..

[17] Inama G., Dodi C., Provini M., Bossoni E., Inama L., Balzarini L., Mancini C., Ramponi S., Marvisi M. (2020). Coronavirus disease 2019 infection in patients with recent cardiac surgery: Does chronic anticoagulant therapy have a protective effect?. J. Cardiovasc. Med. (Hagerstown).

[18] Wang J., Zheng X., Chen J. (2021). Clinical progression and outcomes of 260 patients with severe COVID-19: An observational study. Sci. Rep..

[19] Piva S., Filippini M., Turla F., Cattaneo S., Margola A., De Fulviis S., Nardiello I., Beretta A., Ferrari L., Trotta R. (2020). Clinical presentation and initial management critically ill patients with severe acute respiratory syndrome coronavirus 2 (SARS-CoV-2) infection in Brescia, Italy. J. Crit. Care.

[20] Kim E.J., Lee Y.H., Park J.S., Lee J., Lee S.Y., Kim Y., Kwon Y.S., Jang J.G., Shin K.-C., Kim K.C. (2021). Clinical features and prognostic factors of critically ill patients with COVID-19 in Daegu, South Korea: A multi-center retrospective study. Medicine (Baltim.).

[21] Hong K.S., Lee K.H., Chung J.H., Shin K.C., Choi E.Y., Jin H.J., Jang J.G., Lee W., Ahn J.H. (2020). Clinical Features and Outcomes of 98 Patients Hospitalized with SARS-CoV-2 Infection in Daegu, South Korea: A Brief Descriptive Study. Yonsei Med. J..

[22] Khamis F., Al-Zakani I., Al Naamani H., Al Lawati S., Pandak N., Omar M.B., Al Bahrani M., Bulushi Z.A., Al Khalili H., Al Salmi I. (2020). Clinical characteristics and outcomes of the first 63 adult patients hospitalized with COVID-19: An experience from Oman. J. Infect. Public Health.

[23] Nachega J.B., Ishoso D.K., Otokoye J.O., Hermans M.P., Machekano R.N., Sam-Agudu N.A., Bongo-Pasi Nswe C., Mbala-Kingebeni P., Madinga J.N., Mukendi S. (2020). Clinical Characteristics and Outcomes of Patients Hospitalized for COVID-19 in Africa: Early Insights from the Democratic Republic of the Congo. Am. J. Trop. Med. Hyg..

[24] Medetalibeyoğlu A., Şenkal N., Çapar G., Köse M., Tükek T. (2020). Characteristics of the initial patients hospitalized for COVID-19: A single-center report. Turk. J. Med. Sci..

[25] Ferguson J., Rosser J.I., Quintero O., Scott J., Subramanian A., Gumma M., Rogers A., Kappagoda S. (2020). Characteristics and Outcomes of Coronavirus Disease Patients under Nonsurge Conditions, Northern California, USA, March-April 2020. Emerg. Infect. Dis..

[26] Elabbadi A., Turpin M., Gerotziafas G.T., Teulier M., Voiriot G., Fartoukh M. (2021). Bacterial co-infection in critically ill COVID-19 patients with severe pneumonia. Infection.

[27] Contou D., Claudinon A., Pajot O., Micaëlo M., Longuet Flandre P., Dubert M., Cally R., Logre E., Fraissé M., Mentec H. (2020). Bacterial and viral co-infections in patients with severe SARS-CoV-2 pneumonia admitted to a French ICU. Ann. Intensive Care.

[28] Dubernet A., Larsen K., Masse L., Allyn J., Foch E., Bruneau L., Maillot A., Lagrange-Xelot M., Thomas V., Jaffar-Bandjee M.-C. (2020). A comprehensive strategy for the early treatment of COVID-19 with azithromycin/hydroxychloroquine and/or corticosteroids: Results of a retrospective observational study in the French overseas department of Réunion Island. J. Glob. Antimicrob. Resist..

[29] Kolenda C., Ranc A.-G., Boisset S., Caspar Y., Carricajo A., Souche A., Dauwalder O., Verhoeven P.O., Vandenesch F., Laurent F. (2020). Assessment of Respiratory Bacterial Coinfections Among Severe Acute Respiratory Syndrome Coronavirus 2-Positive Patients Hospitalized in Intensive Care Units Using Conventional Culture and BioFire, FilmArray Pneumonia Panel Plus Assay. Open Forum Infect. Dis..

[30] Movahed S.M.M., Akhavizadegan H., Dolatkhani F., Nejadghaderi S.A., Aghajani F., Gangi M.F., Ghazi Z., Ghasemi H. (2021). Different incidences of acute kidney injury (AKI) and outcomes in COVID-19 patients with and without non-azithromycin antibiotics: A retrospective study. J. Med. Virol..

[31] Zuccon W., Comassi P., Adriani L., Bergamaschini G., Bertin E., Borromeo R., Corti S., De Petri F., Dolci F., Galmozzi A. (2021). Intensive care for seriously ill patients affected by novel coronavirus Sars-CoV-2: Experience of the Crema Hospital, Italy. Am. J. Emerg. Med..

[32] Zhang L., Huang B., Xia H., Fan H., Zhu M., Zhu L., Zhang H., Tao X., Cheng S., Chen J. (2020). Retrospective analysis of clinical features in 134 coronavirus disease 2019 cases. Epidemiol. Infect..

[33] Jamous F., Meyer N., Buus D., Ateeli H., Taggart K., Hanson T., Alzoubaidi M., Nazir J., Devasahayam J. (2020). Critical Illness Due to Covid-19: A Description of the Surge in a Single Center in Sioux Falls. S. D. Med..

[34] Al-Matary A., Almatari F., Al-Matary M., AlDhaefi A., Alqahtani M.H.S., Alhulaimi E.A., AlOtaiby S., Almehiny K., John L.S., Alanazi F.S. (2021). Clinical outcomes of maternal and neonate with COVID-19 infection–Multicentre study in Saudi Arabia. J. Infect. Public Health.

[35] Arshad S., Kilgore P., Chaudhry Z.S., Jacobsen G., Wang D.D., Huitsing K., Brar I., Alangaden G.J., Ramesh M.S., McKinnon J.E. (2020). Treatment with hydroxychloroquine, azithromycin, and combination in patients hospitalized with COVID-19. Int. J. Infect. Dis..

[36] Alfraij A., Bin Alamir A.A., Al-Otaibi A.M., Alsharrah D., Aldaithan A., Kamel A.M., Almutairi M., Alshammari S., Almazyad M., Macarambon J.M. (2021). Characteristics and outcomes of coronavirus disease 2019 (COVID-19) in critically ill pediatric patients admitted to the. intensive care unit: A multicenter retrospective cohort study. J. Infect. Public Health.

[37] Lagier J.-C., Million M., Gautret P., Colson P., Cortaredona S., Giraud-Gatineau A., Honoré S., Gaubert J.-Y., Fournier P.-E., Tissot-Dupont H. (2020). Outcomes of 3737 COVID-19 patients treated with hydroxychloroquine/azithromycin and other regimens in Marseille, France: A retrospective analysis. Travel Med. Infect. Dis..

[38] Bowes E., Joslin J., Braide-Azikiwe D.C.B., Tulley C., Bramham K., Saha S., Jayawardene S., Shakoane B., Wilkins C.J., Hutchings S. (2021). Acute Peritoneal Dialysis with Percutaneous Catheter Insertion for COVID-19–Associated Acute Kidney Injury in Intensive Care: Experience from a UK Tertiary Center. Kidney Int. Rep..

[39] Lei S., Jiang F., Su W., Chen C., Chen J., Mei W., Zhan L.-Y., Jia Y., Zhang L., Liu D. (2020). Clinical characteristics and outcomes of patients undergoing surgeries during the incubation period of COVID-19 infection. EClinicalMedicine.

[40] Abenza-Abildúa M.J., Ramírez-Prieto M.T., Moreno-Zabaleta R., Arenas-Valls N., Salvador-Maya M.A., Algarra-Lucas C., Rojo Moreno-Arrones B., Sánchez-Cordón B., Ojeda-Ruíz de Luna J., Jimeno-Montero C. (2020). Neurological complications in critical patients with COVID-19. Neurología (Engl. Ed.).

[41] García-Posada M., Aruachan-Vesga S., Mestra D., Humánez K., Serrano-Coll H., Cabrales H., Faccini Á., Mattar S. (2021). Clinical outcomes of patients hospitalized for COVID-19 and evidence-based on the pharmacological management reduce mortality in a region of the Colombian Caribbean. J. Infect. Public Health.

[42] McCarthy C.P., Murphy S., Jones-O’Connor M., Olshan D.S., Khambhati J.R., Rehman S., Cadigan J.B., Cui J., Meyerowitz E.A., Philippides G. (2020). Early clinical and sociodemographic experience with patients hospitalized with COVID-19 at a large American healthcare system. EClinicalMedicine.

[43] Zuo T., Zhan H., Zhang F. (2020). Alterations in Fecal Fungal Microbiome of Patients With COVID-19 During Time of Hospitalization until Discharge. Gastroenterology.

[44] Mustafa L., Tolaj I., Baftiu N., Fejza H. (2021). Use of antibiotics in COVID-19 ICU patients. J. Infect. Dev. Ctries..

[45] Dewi R., Kaswandani N., Karyanti M.R., Setyanto D.B., Pudjiadi A.H., Hendarto A., Djer M.M., Prayitno A., Yuniar I., Indawati W. (2021). Mortality in children with positive SARS-CoV-2 polymerase chain reaction test: Lessons learned from a tertiary referral hospital in Indonesia. Int. J. Infect. Dis..

[46] Al-Hadidi S.H., Alhussain H., Abdel Hadi H., Johar A., Yassine H.M., Al Thani A.A., Eltai N.O. (2021). The Spectrum of Antibiotic Prescribing During COVID-19 Pandemic: A Systematic Literature Review. Microb. Drug Resist..

[47] Buheji M., Nassef M., Shorrab A., Buhiji A., Abosamak M. (2021). Alleviation of Antimicrobial Therapy in ICU during COVID-19 second wave—A Review Paper. Int. J. Manag..

[48] Guan W., Ni Z., Hu Y., Liang W., Ou C., He J., Liu L., Shan H., Lei C., Hui D.S.C. (2020). Clinical characteristics of 2019 novel coronavirus infection in China. medRxiv.

[49] Haas L.E.M., de Lange D.W., van Dijk D., van Delden J.J.M. (2020). Should we deny ICU admission to the elderly? Ethical considerations in times of COVID-19. Crit. Care.

[50] Peckham H., de Gruijter N.M., Raine C., Radziszewska A., Ciurtin C., Wedderburn L.R., Rosser E.C., Webb K., Deakin C.T. (2020). Male sex identified by global COVID-19 meta-analysis as a risk factor for death and ITU admission. Nat. Commun..

[51] Muniyappa R., Gubbi S. (2020). COVID-19 pandemic, coronaviruses, and diabetes mellitus. Am. J. Physiol. Endocrinol. Metab..

[52] Alsadhan I., Alruwashid S., Alhamad M., Alajmi S., Alshehri S., Alfadhli E., Ekhzaimy A. (2020). Diabetic ketoacidosis precipitated by Coronavirus disease 2019 infection: Case series. Curr. Ther. Res..

[53] Bansal M. (2020). Cardiovascular disease and COVID-19. Diabetes Metab. Syndr. Clin. Res. Rev..

[54] Radke R.M., Frenzel T., Baumgartner H., Diller G.-P. (2020). Adult congenital heart disease and the COVID-19 pandemic. Heart.

[55] Qiu H., Wander P., Bernstein D., Satapathy S.K. (2020). Acute on chronic liver failure from novel severe acute respiratory syndrome coronavirus 2 (SARS-CoV-2). Liver Int..

[56] Trujillo H., Caravaca-Fontán F., Sevillano Á., Gutiérrez E., Fernández-Ruiz M., López-Medrano F., Hernández A., Aguado J.M., Praga M., Andrés A. (2020). Tocilizumab use in Kidney Transplant Patients with COVID-19. Clin. Transplant..

[57] Adapa S., Chenna A., Balla M. (2020). COVID-19 Pandemic Causing Acute Kidney Injury and Impact on Patients with Chronic Kidney Disease and Renal Transplantation. J. Clin. Med. Res..

[58] Fiolet T., Guihur A., Rebeaud M.E., Mulot M., Peiffer-Smadja N., Mahamat-Saleh Y. (2021). Effect of hydroxychloroquine with or without azithromycin on the mortality of coronavirus disease 2019 (COVID-19) patients: A systematic review and meta-analysis. Clin. Microbiol. Infect..

[59] Kumar J., Jain S., Meena J., Yadav A. (2021). Efficacy and safety of hydroxychloroquine/chloroquine against SARS-CoV-2 infection: A systematic review and meta-analysis. J. Infect. Chemother..

[60] World Health Organization (WHO) Clinical Management of COVID-19. https://www.who.int/publications/i/item/WHO-2019-nCoV-clinical-2021-1..

[61] Chang R., Elhusseiny K.M., Yeh Y.-C., Sun W.-Z. (2021). COVID-19 ICU and mechanical ventilation patient characteristics and outcomes-A systematic review and meta-analysis. PLoS ONE.

[62] Cravedi P., Mothi S.S., Azzi Y., Haverly M., Farouk S.S., Pérez-Sáez M.J., Redondo-Pachón M.D., Murphy B., Florman S., Cyrino L.G. (2020). COVID-19 and kidney transplantation: Results from the TANGO International Transplant Consortium. Am. J. Transplant..

[63] Vaughn V.M., Gandhi T.N., Petty L.A., Patel P.K., Prescott H.C., Malani A.N., Ratz D., McLaughlin E., Chopra V., Flanders S.A. (2021). Empiric Antibacterial Therapy and Community-onset Bacterial Coinfection in Patients Hospitalized With Coronavirus Disease 2019 (COVID-19): A Multi-hospital Cohort Study. Clin. Infect. Dis..

[64] Chen N., Zhou M., Dong X., Qu J., Gong F., Han Y., Qiu Y., Wang J., Liu Y., Wei Y. (2020). Epidemiological and clinical characteristics of 99 cases of 2019 novel coronavirus pneumonia in Wuhan, China: A descriptive study. Lancet.

[65] Nag V.L., Kaur N. (2021). Superinfections in COVID-19 Patients: Role of Antimicrobials. Dubai Med. J..

[66] MacIntyre C.R., Chughtai A.A., Barnes M., Ridda I., Seale H., Toms R., Heywood A. (2018). The role of pneumonia and secondary bacterial infection in fatal and serious outcomes of pandemic influenza a (H1N1)pdm09. BMC Infect. Dis..

[67] Lansbury L., Lim B., Baskaran V., Lim W.S. (2020). Co-infections in people with COVID-19: A systematic review and meta-analysis. J. Infect..

[68] Borgogna T.R., Hisey B., Heitmann E., Obar J.J., Meissner N., Voyich J.M. (2018). Secondary Bacterial Pneumonia by Staphylococcus aureus Following Influenza a Infection Is SaeR/S Dependent. J. Infect. Dis..

[69] Duployez C., Le Guern R., Tinez C., Lejeune A.-L., Robriquet L., Six S., Loïez C., Wallet F. (2020). Panton-Valentine Leukocidin–Secreting Staphylococcus aureus Pneumonia Complicating COVID-19. Emerg. Infect. Dis. J..

[70] Yap F.H.Y., Gomersall C.D., Fung K.S.C., Ho P.-L., Ho O.-M., Lam P.K.N., Lam D.T.C., Lyon D.J., Joynt G.M. (2004). Increase in Methicillin-Resistant Staphylococcus aureus Acquisition Rate and Change in Pathogen Pattern Associated with an Outbreak of Severe Acute Respiratory Syndrome. Clin. Infect. Dis..

[71] Rees E.M., Nightingale E.S., Jafari Y., Waterlow N.R., Clifford S.B., Pearson C.A., Group C.W., Jombart T., Procter S.R., Knight G.M. (2020). COVID-19 length of hospital stay: A systematic review and data synthesis. BMC Med..

[72] Lewnard J.A., Liu V.X., Jackson M.L., Schmidt M.A., Jewell B.L., Flores J.P., Jentz C., Northrup G.R., Mahmud A., Reingold A.L. (2020). Incidence, Clinical Outcomes, and Transmission Dynamics of Hospitalized 2019 Coronavirus Disease among 9,596,321 Individuals Residing in California and Washington, United States: A Prospective Cohort Study. medRxiv.

[73] Richardson S., Hirsch J.S., Narasimhan M., Crawford J.M., McGinn T., Davidson K.W. (2020). Presenting Characteristics, Comorbidities, and Outcomes Among 5700 Patients Hospitalized With COVID-19 in the New York City Area. JAMA.

[74] Moher D., Liberati A., Tetzlaff J., Altman D.G., Group T.P. (2009). Preferred Reporting Items for Systematic Reviews and Meta-Analyses: The PRISMA Statement. PLOS Med..

