# Supplementary material for: Antibiotics Prescribing in Intensive Care Settings during the COVID-19 Era: A Systematic Review
Source: Antibiotics (Basel). 2021 Aug 2;10(8):935. doi: 10.3390/antibiotics10080935 (PMC8389042; doi:10.3390/antibiotics10080935)
Supplement: Supplementary file 1 [file antibiotics-10-00935-s001.zip › antibiotics-1269445-supplementary.pdf]

antibiotics

Table S1. Demographical and clinical data of 38 studies related to antibiotics perscription in the ICU settings for COVID19 patients.

Authors, Year	Country	No. of ICU Patients	No. of ICU Patients Received Antibiotics	Age Median (IQR)[1]	Gender	Antibiotics Prescribed [No. (%)]	Comorbidities [No. (%)]	Infection Type	Bacterial Infection in ICU Patients	Death Rate [No. (%)][2]	ICU Stay Length Median (IQR) Days
Shekerdemian, Lara S., et al., 2020 [8]	USA	48	8	13 (4.2-16.6)	Male: 25	Azithromycin: 8 (17)	Immune suppression/malignancy: 11(23)<br>Obesity: 7 (15)<br>Diabetes: 4 (8)<br>Seizures: 3 (6)<br>Congenital heart disease: 3 (6)<br>Sickle cell disease: 2 (4)<br>Chronic lung disease: 3 (4)<br>Other congenital malformation: 2 (4)	NR[1]	NR	NR	5 (3–9)
Faqihi, Fahad, et al., 2020 [9]	KSA	10	10	51 (45.1–55.9)	Male: 7	Broadspectrum antibiotics: 10 (100)	Diabetes mellitus: (60)<br>Essential hypertension: (50)<br>Cardiovascular disease: (10)	Secondary Infection	*Gram Negative*	NR	15 (13.2–19.6)
Wang, Rongrong, et al., 2021 [10]	China	33	22	NR	NR	Antibiotics: 22 (100)	Hypertension: 16<br>Diabetes: 5<br>Cardiac disease: 3<br>Others: 4	NR	NR	NR	NR
Lima-Setta, Fernanda, et al., 2020 [11]	Brazil	56	33	6.2 (1months-19 years)	Male: 39<br>Female: 17	Antibiotics: 59 (33)	Chronic neurological disease: 5 (45.5)<br>Asthma: 1 (9.1)<br>Congenital heart defect: 2 (18.2)<br>Undernutrition: 1 (9.1)<br>Obesity: 1 (9.1)<br>Diabetes: 1 (9.1)<br>Adrenoleukodys trophy (ADL): 1 (9.1)	NR	NR	1 (1.8)	6 (5–11)
Maataoui, Naouale, et al., 2021[12]	France	191	53	57 (46-65)	Male: 55	Penicillins<br>Amoxicillin clavulanate<br>3rd generation cephalosporin<br>4th generation cephalosporin<br>Piperacillin tazobactam<br>Carbapenems<br>Others	Diabetes: 19 (28)<br>Renal failure: 14 (21)<br>Respiratory failure: 18 (27)<br>Heart failure: 23 (34)<br>Smoking: 4 (6)<br>Alcoholism: 3 (4)<br>Hypertension: 32 (48)<br>Transplants: 9 (13)<br>Cancer: 2 (3)	Co-infection	*Haemophilus influenza: 1*<br>*Pseudomonas aeruginosa: 12*<br>*Staphylococcus aureus: 10*<br>*Escherichia coli: 9*<br>*Klebsiella spp.: 14*<br>*Acinetobacter baumannii: 4*<br>Others *in HAP/VAP: 12*<br>*Stenotrophomonas maltophilia: 3*<br>*Morganella morganii: 1*<br>*Burkholderia gladioli: 1*	38 (56)	NR

Sharifipour, Ehsan, et al., 2020 [13]	Iran	19	19	67 [mean] (38-92)	Male: 11<br>Female: 8	Ceftriaxone: 19 (100)<br>Azithromycin: 19 (100)<br>**before admission to the ICUs**	Kidney disease<br>Diabetes<br>Hypertension<br>Heart disease	Co-infection	*Acinetobacter baumannii: 17*<br>*Staphylococcus aureus: 2*<br>*MRSA: 1*<br>*MSSA: 1*	18 (95)	15
Colmenero, Jordi, et al., 2021 [14]	Spain	12	12	68.51 [mean]	NR	azithromycin: 12 (100)	Diabetes<br>Hypertension<br>ACE inhibitors Cardiomyopathy<br>Bronchopulmonary	NR	NR	NR	NR
Yang, Luhuan, et al., 2020 [15]	China	29	29	71 [mean]	Male: 16<br>Female: 13	Moxifloxacin: 29 (100)<br>Ceftriaxone: 29 (100)	Hypertension: 9 (31)<br>Chronic lung disease: 4 (13.8)<br>Diabetes: 4 (13.8)<br>Chronic heart disease: 1 (3.4)<br>Chronic kidney disease: 2 (6.9)<br>Malignancy: 1 (3.4)	NR	NR	15 (51.7)	NR
Pereira, Marcus R., et al., 2020 [16]	USA	23	19	67 (56-74)	NR	Azithromycin: 19 (82.6)	NR	NR	NR	16 (69.6)	NR
Inama, Giuseppe, et al., 2020[17]	Italy	1	1	85 years	Female	Azithromycin: 1 (100)	Hypertension: 1 (100)<br>Dyslipidemia: 1 (100)<br>Atrial fibrillation; CAD: 1 (100) Coronary artery disease: 1 (100)<br>Chronic obstructive pulmonary disease: 1 (100)	NR	NR	1 (100)	NR
Wang, Junjuan, et al., 2020 [18]	China	17	17	NR	NR	Cephalosporins (Sulperazone)<br>Quinolones<br>Carbapenems<br>Tigecycline	Respiratory failure septic shock<br>Cardiovascular disease<br>Cerebrovascular disease<br>Digestive system disease<br>Diabetes<br>Chronic renal insufficiency<br>Respiratory system disease<br>Nervous system disease<br>Malignant tumor<br>Rheumatism and connective tissue disease	Co-infection	*Acinetobacter baumannii: 10 Klebsiella pneumonia: 17*<br>*Mycoplasma pneumonia: 3*	NR	NR
Piva, Simone, et al., 2020 [19]	Italy	33	33	64 (59–72)	Male: 30<br>Female: 3	Carbapenem: 33 (100)<br>Cephalosporin: 33 (100) Macrolide: 33 (100)<br>Penicillin: 33 (100)	Heart disease: 14 (43)<br>Obesity: 6 (18)<br>Lung disease: 4 (12)<br>Diabetes: 2 (6)<br>Autoimmune disease or immunodeficiency:1 (3)	Co-infection	*Extended spectrum beta-lactamase E. coli*	1 (3.03)	NR
Kim, Eun Jin, et al., 2021 [20]	South Korea	110	110	71 (63-78)	Male: 67<br>Female: 43	Antibiotics: 110 (100)	Hypertension: 55 (50)<br>Diabetes: 40 (36.4)<br>Chronic kidney disease: 11 (10)<br>Dementia: 11 (10)<br>Cerebrovascular disease: 10(9.1)<br>Malignancy: 10 (9.1)	Co-infection	*Pneumococcus pneumonia*<br>*Mycoplasma pneumonia*<br>*Pseudomonas aeruginosa*	47 (42.7)	28.5 (15.8–42.0)

							Cardiovascular disease: 9 (8.2)<br>Chronic lung disease: 9 (8.2)<br>Chronic liver disease: 5 (4.5)				
Hong, Kyung Soo, et al., 2020 [21]	South Korea	13	13	(21–49):1<br>(50–64): 6<br>(≥65): 6	Male: 6<br>Female: 7	Antibiotics: 13 (100)	Diabetes mellitus: 3<br>Hypertension: 5<br>Malignancy: 1<br>Parkinson's disease: 1	NR	NR	4 (30.8)	1.2±1.5 [Mean]
Khamis, Faryal, et al., 2020 [22]	Oman	24	24	50 [Mean]	Male: 21<br>Female: 3	Ceftriaxone: 16 (67)<br>Azithromycin: 13 (54)<br>Piperacillin/ tazobactam combination: 19 (79)<br>Meropenem: 7 (29)	Hypertension: 7 (29)<br>Diabetes mellitus: 11 (46)<br>Heart disease: 2 (8.3)<br>Renal disease: 1 (4.2)	NR	NR	5 (20.8)	8 (5.5–13)
Nachega, Jean B., et al., 2020 [23]	Democratic Republic of the Congo	191	191	58 (44–66)	Male: 135<br>Female: 55<br>1 missing	Azithromycin<br>3rd generation cephalosporin	Hypertension: 87 (45.6)<br>hear disease: 21 (11.1)<br>Obesity: 22 (11.5)<br>Diabetes: 60 (31.6)<br>Asthma/chronic obstructive pulmonary disease: 12 (6.3)<br>Chronic kidney disease: 3 (1.6)<br>Cancer: 3 (1.6)	NR	NR	86 (45)	NR
Medetalibeyoğlu, Alpay, et al., 2020 [24]	Turkey	11	11	70.1 [Mean]	Male: 8<br>Female: 3	Azithromycin: 11 (100)<br>Ceftriaxone: 11 (100)	Diabetes<br>Coronary heart disease<br>Hematologic malignancy<br>Solid malignancy.<br>Chronic obstructive pulmonary disease.	NR	NR	NR	NR
Ferguson, Jessica, et al., 2020 [25]	USA	21	14	57.6 (42.2–70.1)	Male: 13<br>Female: 8	Azithromycin: 14 (66.7)	Acute respiratory distress syndrome: 13 (61.9)<br>Arrhythmia: 6 (28.6)<br>Pneumonia: 5 (23.8)<br>Acute kidney injury: 4 (19.0)<br>Catheter-related bloodstream infection: 2 (9.5)<br>Cardiomyopathy: 2 (9.5)	NR	NR	3 (14.3)	12
Elabbadi, Alexandre, et al., 2021 [26]	France	101	58	61 (53–69)	Male: 79	3rd generation cephalosporin: 58	Arterial hypertension: 66 (65.3)<br>Coronary heart disease: 14 (13.9)<br>Cerebrovascular disease : 7 (6.9)<br>Peripheral artery disease: 4 (4)<br>Previous venous thromboembolism: 3 (3)<br>Diabetes: 33 (32.7)<br>Chronic pulmonary disease: 7 (6.9)<br>Cancer or hematologic malignancy: 4 (4)<br>Chronic kidney disease: 21 (20.8)<br>Chronic dialysis :9 (8.9)	Co-infection	Gram-positive cocci: *12*<br>*Staphylococcus aureus: 11*<br>*MSSA: 9*<br>*MRSA:2*<br>*Streptococcus pneumonia:1*<br>Gram-negative bacilli*: 13*<br>*E. coli: 2*<br>*Klebsiella spp: 2*<br>*Enterobacter, Citrobacter, Hafnia spp: 4*<br>*Pseudomonas spp: 2*	21 (21)	14 (6–26)

Contou, Damien, et al., 2020 [27]	France	92	39	61 (55-70)	Male: 73	Cefotaxime: 14 (36)<br>Amoxicillin/clavulanate combination: 13 (33)<br>Amoxicillin: 6 (15)<br>Piperacillin/tazobactam<br>Combination: 1(3)<br>Others antibiotic: 5 (13)	Obesity: 38<br>Hypertension: 59<br>Diabetes mellitus: 35<br>Cardiovascular diseases: 9<br>Atrial fibrillation: 3<br>Cerebrovascular diseases: 8<br>Chronic respiratory diseases: 18<br>Chronic renal failure: 7<br>Immunocompromised:9	Co-infection	*Staphylococcus aureus: 10 (31)*<br>*Haemophilus influenza: 7 (22)*<br>*Streptococcus pneumonia: 6 (19)*<br>*Enterobacteriaceae: 5 (16)*<br>*Pseudomonas aeruginosa: 2 (6)*<br>*Moraxella catarrhalis: 1 (3)*<br>*Acinetobacter baumannii: 1 (3)*	45 (49)	1 (0–4)
Dubernet, Arthur, et al., 2020 [28]	France	10	1	71 (59–74)	Male: 10	3rd generation cephalosporin: 1<br>Azithromycin: 1	Hypertension: 6<br>Diabetes mellitus: 3<br>Obesity: 2<br>Heart disease: 2<br>Respiratory disease: 2<br>Kidney disease: 3	NR	*NR*	0	25 (15–35) patients treated with HCQ/AZT and 40 (25–55) treated with HCQ/AZT
Kolenda, Camille, et al., 2020 [29]	France	99	72	NR	NR	Amoxicillin and clavulanic acid: 72<br>3rd generation cephalosporins: 72	Acute respiratory syndrome	Co-infection	*Staphylococcus aureus: 7*<br>*Haemophilus influenza: 4*<br>*Streptococcus pneumonia: 2*<br>*Enterobacteriaceae: 2*<br>*Moraxella catarrhalis: 1*<br>*Legionella pneumophila: 1*	NR	NR
Movahed, Seyed Majid Mousavi, et al., 2021 [30]	Iran	183	120	NR	NR	NR	NR	NR	NR	79 (43.2)	NR
Zuccon, William, et al., 2020 [31]	Italy	54	54	(30-89)	Male: 44<br>Female: 10	NR	Renal failure: 16<br>Peritoneal dialysis: 10<br>Liver failure: 7<br>Lesions due to ventilation: 6<br>Heart disease: 3<br>Central neurological: 5<br>Meningo-encephalitis: 2<br>Cerebral haemorrhage: 1<br>peripheral neurological event: 2<br>Paresis: 3	NR	NR	21 (38.9)	28 [Average] (1 – 56)
Zhang, Lin, et al., 2020 [32]	China	134	131	60.78 [Mean]	Male: 87<br>Female: 47	Fluoroquinolones: 131 (97.76)<br>Carbapenems:131 (97.76)	Acute Respiratory Distress Syndrome: 105 (78.36)<br>Acute cardiac injury: 75 (55.97)<br>Pneumothorax: 3 (2.24)<br>Liver dysfunction: 53 (39.55)<br>Acute kidney injury: 63 (47.01)	NR	NR	101 (75.4)	10 (8–13)

Jamous, Fady, et al., 2020 [33]	USA	37	37	NR	NR	NR	Diabetes: (43)<br>Hypertension: (47)	NR	NR	NR	11
Al-Matary, Abdulrahman, et al., 2021 [34]	KSA	86	62	Neonate	NR	NR	NR	NR	NR	0	8 (13–7)
Arshad, Samia, et al., 2020 [35]	USA	614	309	NR	NR	Azithromycin	NR	NR	NR	460 (74.9)	(2.3 - 5.3)
Alfraij, Abdulla, et al., 2021 [36]	KSA	25	25	2.78 (0.21-8.51)	Male: 15	Penicillins: 4 (16)<br>Third-generation cephalosporins: 14 (56)<br>Macrolides: 4 (16)<br>Lincosamides: 3 (12)<br>Beta lactam-beta lactamase inhibitor (anti-pseudomonal): 11 (44)<br>Glycopeptides: 11 (44)<br>Carbapenems: 5 (20)<br>Aminoglycosides: 2 (8)<br>Trimethoprim-sulfonamide combinations: 2 (8)<br>Fluroquinolones: 1 (4)<br>Imidazoles: 1 (4.2)	Neurological disease: 6 (24)<br>Hematological malignancies: 5 (20)<br>Congenital heart disease: 4 (16)	NR	NR	4 (16)	5 (3.75–21)
Lagier, Jean-Christophe, et al., 2020 [37]	France	67	64	NR	NR	Azithromycin	NR	Co-infection	*Streptococcus pneumoniae*<br>*Staphylococcus aureus*<br>*Morganella morganii,*<br>*Enterobacter cloacae*<br>*Stenotrophomonas maltophilia,*<br>*Enterobacter cloacae*<br>*Klebsiella pneumoniae*<br>*Staphylococcus aureus*<br>*Escherichia coli*	35(52.2)	NR

Bowes, Elaine, et al., 2021 [38]	UK	44	44	60 (55-64)	Male: 35	Vancomycin<br>Broad-spectrum antibiotics	Diabetes: 20 (45.5)<br>Hypertension: 30 (68.2)<br>Cardiovascular disease: 10 (22.7)	NR	NR	NR	13.5 (10.0–20.3)
Lei, Shaoqing, et al., 2020 [39]	China	15	15	55 (44-74)	Male: 5	Antibiotics	Any comorbidity: 12 (80)<br>Hypertension: 9 (60)<br>Malignancy: 5 (33·3)<br>Diabetes: 6 (40)<br>Cardiovascular disease: 6 (40)<br>Cerebrovascular disease: 2 (13·3)<br>COPD: 1 (6·7) Chronic kidney disease: 1 (6·7)	NR	NR	7 (46.7)	NR
Abenza-Abildúa, M. J., et al., 2020 [40]	Spain	30	30	57.41	Male: 21	Ceftriaxone<br>Empirical Antibiotic	Arterial hypertension: 8 (26.66)<br>Obesity: 8 (26.66)<br>Diabetes mellitus: 6 (20)	NR	NR	3 (10)	18.83 [Mean] (6-55)
García-Posada, Mara, et al., 2021 [41]	Colombia	100	70	NR	NR	Cefepime<br>Piperacillin<br>Tazobactam<br>Meropenem<br>Vancomycin	NR	Co-infection	*Klebsiella pneumonia*<br>*Pseudomonas aeruginosa*<br>*Staphylococcus aureus*<br>*Enterobacter cloacae*	NR	NR
McCarthy et al., 2020 [42]	USA	112	110	63.5 (52.5-77.3)	Male: 71<br>Female: 41	Azithromycin<br>Other antibiotics	Obese: 47 (42)<br>Asthma: 11 (9.8)<br>COPD: 10 (8.9)<br>Interstitial lung disease: 1 (0.9)<br>OSA/OHS: 14 (12.5)<br>Hypertension: 63 (56.2)<br>Hyperlipidemia: 55 (49.1)<br>Diabetes Mellitus: 36 (32.1)<br>Known CAD: 16 (14.3)<br>Prior MI: 6 (5.4)<br>Prior revascularization: 10 (8.9)<br>Heart Failure: 12 (10.7)<br>PAD: 2 (1.8)<br>Prior stroke/TIA: 3 (2.7)<br>Atrial fibrillation: 16 (14.3)<br>Liver cirrhosis: 2 (1.8)<br>CKD: 11 (9.8)<br>CKD on dialysis: 5 (4.5)<br>History of malignancy: 22 (19.6)<br>Active malignancy: 11 (8.9)<br>Prior organ transplant: 4 (3.6)<br>Rheumatologic or inflammatory disorder requiring immunosuppression: 9 (8)	NR	NR	3 (2.68)	15 (5-24.5)
Zuo, Tao, et al., 2020 [43]	China	2	1	Male: 42<br>Female: 65	Male: 1<br>Female: 1	Daptomycin<br>Meropenem	Female : Hypothyroidism<br>Hypertension<br>Chronic hepatitis B carrier	NR	NR	0	NR

Mustafa, Lirim, et al., 2021 [44]	Kosovo	52	52	61 (20-86)	Male: 35	Mipenem: 30 (58)<br>Ceftriaxone: 28 (54)<br>Fluoroquinolones: 22 (42)<br>Piperacillin/tazobactam: 17 (33)<br>Ceftazidime: 3 (6)<br>Meropenem: 1 (2)	Obesity: 23 (44)<br>Hypertension: 9 (17)<br>Diabetes mellitus: 21 (40)<br>Cardio-vascular diseases: 8 (15)<br>Atrial fibrillation: 10 (19)<br>Cerebrovascular diseases: 8 (15)<br>Venous thromboembolism: 8 (15)<br>Chronic respiratory diseases: 17 (33)<br>Chronic renal failure: 14 (27)<br>Cerebrovascular diseases: 8 (15)	Co-infection	MRSA	29 (55.7)	NR
Dewi, Rismala, et al., 2021 [45]	Indonesia	16	16	NR	NR	Antibiotics (not spacified)	NR	NR	NR	16 (100)	1.5 (0–11)

[1] Abbreviation: IQR: Interquartile Range, NR: Not Reported, [2] Death Rate = (No. of deaths from ICU patients/Total No. of ICU patients) * 100.
